# Neuromyelitis optica - an update: 2007–2009

**DOI:** 10.4103/0972-2327.58277

**Published:** 2009

**Authors:** Anu Jacob

**Affiliations:** The Walton Centre for Neurology and Neurosurgery, Liverpool, L97LJ, United Kingdom

**Keywords:** Aquaporin, multiple sclerosis, myelitis, neuromyelitis optica

## Abstract

Neuromyelitis optica is an inflammatory demyelinating disorder of the central nervous system. The discovery of a specific antibody (NMO IgG /aquaporin-4 antibody) in patients with this condition has led to a marked revival of research on the disease. This article summarizes the major advances in neuromyelitis optica, particularly in the last 2 years, and supplements the previous review published in this Journal in 2007. Important among these developments are: the epidemiological studies, which have provided estimates of incidence and prevalence; identification of mutations in the aquaporin-4 gene; improved understanding of the effects of anti-aquaporin-4 antibody on astrocytes; roles of excitatory amino acid transporter type 2 and glutamate; requirement of aquaporin-4 to be in orthogonal arrays to be antigenic; recognition of the presence of aquaporin-4 antibody in patients with cancer and posterior reversible encephalopathy syndrome; possibility of monitoring the disease using the antibody, and the effectiveness of rituximab and mycophenolate in preventing relapses.

## Introduction

Neuromyelitis optica (NMO) is an inflammatory demyelinating disease of the central nervous system (CNS), usually affecting the optic nerves and the spinal cord. It is presumed to be an antibody-mediated disorder, the target antigen being the water channel aquaporin-4 (AQP4) on astrocyte cell membranes.[1,2] We recently reviewed the major advances in NMO over the last decade in this Journal.[3] This update summarizes the developments in the last 2 years. To identify articles of interest I used the term ‘neuromyelitis optica’ to conduct a PubMed search from July 2007 to May 2009. Selected recent conference proceedings were also reviewed.

## Epidemiology

NMO is believed to affect women more than men and people of Asian and African ancestry more than Caucasians. A summary of all the reported cases of NMO from India estimated that 9–24% of demyelinating disease fulfilled the criteria for diagnosis of NMO. Pandit *et al*. recently reported that 10% (8/78) of a South Indian cohort of patients with demyelinating disease satisfied the criteria for NMO.[4] However, these are not population-based reports. A recent population-based survey done in 2003–2004, which covered 98% of Cuban population, found a prevalence of 0.51 per 100,000 and an average annual incidence rate of 0.053 per 100,000. Neither of these rates differed significantly between whites, blacks, mixed, or non-whites. The prevalence rates by gender demonstrated a much higher rate in females (0.91) than in males (0.12).[5]

## Clinical Features

NMO is a relapsing disorder (a monophasic disorder is uncommon), with the median age of onset in the fourth decade.[6] The characteristic symptoms of NMO are optic neuritis and longitudinally extensive transverse myelitis (LETM). Brainstem involvement (often manifested as nausea, hiccups, and respiratory failure), paroxysmal tonic spasms, hemiparesis, and stupor can occur. Cerebral symptoms are more common in children. Up to 45% of NMO IgG seropositive children are symptomatic with vomiting and encephalopathy.[7]

A variety of systemic autoimmune disorders and autoantibodies coexist in NMO. These autoimmune diseases include thyroid disease, myasthenia gravis,[8] celiac disease,[9] systemic lupus erythematosis (SLE), and Sjögren's syndrome.[10] If typical optic neuritis and myelitis occur in a patient with SLE or Sjögren's, it is important to look for NMO IgG/AQP-4 antibody as, the co-occurrence of the two disorders can occur and is more likely than SLE or Sjögren syndrome causing myelitis.[10] Non-organ-specific autoantibodies are common in NMO: ANA is seen in 40%, SSA in 16%, and neuronal or muscle acetylcholine receptor antibodies in 38.5%.[11]

Relapses in NMO develop quickly over days and can leads to rapid paraparesis or markedly impaired vision. Incomplete recovery from disability acquired during even initial relapses is common, in contrast to the typical relapsing MS where disability accumulates during the progressive phase many years from onset. Mortality rates in untreated NMO are high.

In the largest reported series, more than half of the patients developed severe visual loss in at least one eye or had inability to ambulate without assistance within 5 years of disease onset.[6] The 5-year mortality rate in relapsing patients was 32%. Prospective studies from the United Kingdom show that at a median follow-up of 3 years after recruitment into the study, 23% (10 out of 42) of the patients died.[12]

## AQP4 4–negative NMO

There seems to be no major clinical differences between the AQP4-Ab negative and positive relapsing groups.[1] In Japanese cases, the frequency and severity of relapses seem less in antibody-negative patients.[13] Only 12.5% of monophasic NMO patients are antibody positive, whereas 80% of patients with relapsing NMO are seropositive.

## Diagnostic Criteria

The diagnostic criteria of NMO have evolved over decades. The present criteria for diagnosis specifies optic neuritis and acute myelitis and at least two of three supportive criteria, (a contiguous spinal cord MRI lesion extending over three vertebral segments, brain MRI not meeting the diagnostic criteria for multiple sclerosis, and NMO-IgG/AQP4 antibody seropositive status).[14]

When there is isolated or recurrent optic neuritis or transverse myelitis along with the presence of AQP4 antibody the patient is said to have an NMO spectrum disorder. Such presentations are being increasingly recognised and treated as NMO.[15] Untreated, such patients relapse and seem to behave like typical NMO.[16,17] As the availability of antibody tests for NMO increases, the diagnosis and treatment of such disorders should occur earlier and, hopefully, typical NMO in its full form (i.e., with optic neuritis and myelitis) will become less common.

## Differential Diagnosis of NMO

Typical NMO is ‘hard to miss,’ with episodes of severe optic neuritis and myelitis accompanied by a longitudinally extensive (more than three vertebral segments) spinal cord signal and the absence of oligoclonal bands. The commonest misdiagnosis is MS (which can be differentiated by the typical brain MRI picture, short segment spinal cord lesions, presence of oligoclonal bands, and good recovery from relapses). Other disorders that could have an acute opticospinal presentation include acute disseminated encephalomyelitis, lymphoma, SLE, Sjögren's syndrome, and herpes zoster.[18] CRMP-5/antiCV-2 antibody in association with systemic cancers have been found in patients with an NMO-like presentation.[19,20] The differential diagnoses of isolated or relapsing optic neuritis or transverse myelitis includes numerous conditions and are summarized elsewhere.[21,22]

## Neuroimaging

Spinal cord MRI shows a T2-hyperintense, longitudinally extensive (involving more than three vertebral segments) lesion during an acute episode of myelitis. MS can very rarely mimic this appearance, e.g., coalescence of multiple plaques can appear as one long lesion. However, in 10% of children with MS, myelitis can be truly ‘longitudinally extensive.’[23] Spinal cord lesions in NMO can resemble the changes seen in anterior spinal artery stroke.[24]

**Figure 1:**
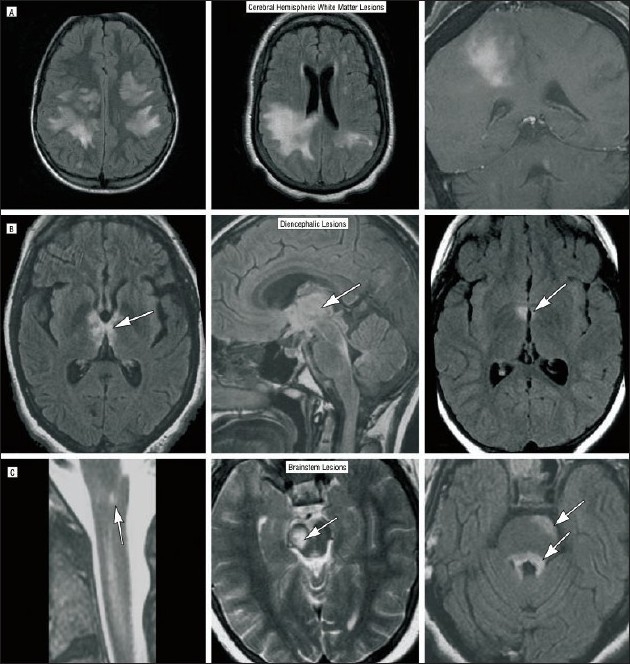
Atypical brain lesions encountered in patients with NMO as seen on MRI. (A, left) Extensive bihemispheric, subcortical, nonenhancing white matter fluid-attenuated inversion recovery (FLAIR) signal abnormality. (A, center) A large confluent FLAIR signal abnormality in the right parietal area that demonstrates diffuse gadolinium enhancement (A, right). (B) FLAIR abnormality in the hypothalamus (B, right, arrow) and the right cerebral peduncle (C, center, arrow). (B, center) FLAIR signal abnormality in the thalamus (arrow), hypothalamus, and optic chiasm, extending into the superior cerebellar peduncle and the floor of the fourth ventricle. (B, left) A confluent nonenhancing signal abnormality extending from the anterosuperior thalamus-hypothalamus (arrow) to the optic tracts behind the chiasm to the superior surface of the mesencephalon extending to the periaqueductal area (right, left) to the superior cerebellar peduncles, and the pontine tegmentum (C, right, arrows). Extension of T2-weighted MRI signal abnormality into the medulla (C, left, arrow) in a patient with an otherwise normal brain MRI. Reprinted with permission from Sean Pittock (Mayo Clinic Rochester, USA) and Arch Neurol. 2006 Mar; 63(3): 390-6(25). Copyright American Medical Association, 2003, American Medical Association, All rights reserved.

**Figure 2:**
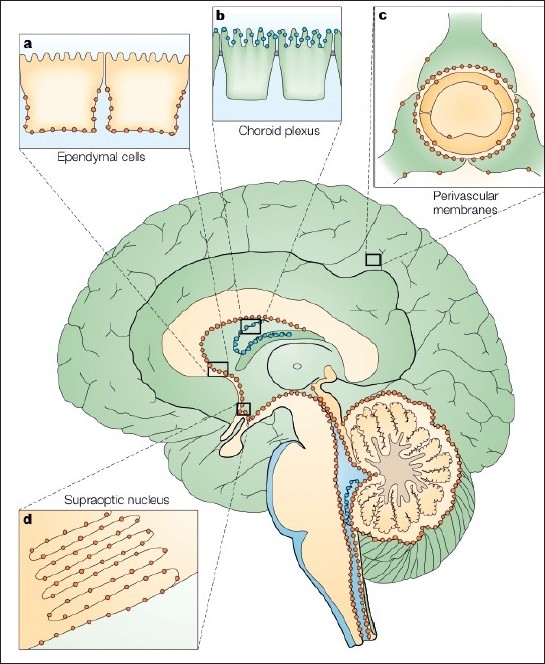
Aquaporin 4 in the brain. Several AQP4 subtypes are present in the brain. The distribution in the brain of AQP1 (blue) and AQP4 (orange) is schematically illustrated on a sagittal section of a human brain. The brain AQP shows four different expression patterns (a–d). (a) AQP4 occurs in the basolateral membrane of ependymal cells; (b) AQP1 is expressed at the apical membrane of the choroid plexus epithelial cells; (c) AQP4 is concentrated in the astrocytic end-feet, specifically in those membrane domains that abut on brain capillaries or on the pia; (d) AQP4 is expressed in glial lamellae of the supraoptic nucleus and other osmosensitive regions. AQP4 also occurs in non-end-feet membranes of astrocytes, but at comparatively low concentrations. They are absent from neurons, oligodendrocytes, and microglia. Reprinted with permission from OP Ottersen and Macmillan Publishers Ltd: The Molecular Basis of Water Transport in the Brain. Nat Rev Neurosci. 2003 Dec;4(12):991-1001. (26), Copyright (2003)

Brain lesions can occur in up to 60% of patients and are usually subtle but can sometimes be prominent[25] [Figure 1]. They seem to predominate in areas with high AQP4 expression[26] [Figure 2]. The lesions are usually asymptomatic but may cause symptoms that may include lethargy, endocrine dysfunction, Parinaud syndrome,[27] hemiparesis, and coma. Large callosal lesions have been described in NMO.[28] Spectroscopy has shown that unaffected white matter (normal-appearing white matter; NAWM) has a normal Naa/Cr in patients with NMO in contrast to those with MS.[29] Optical coherence tomography (a technique for obtaining subsurface images of tissue morphology at much higher resolution than can be obtained with other imaging modalities such as MRI or ultrasound) has shown that the nerve fiber layer thickness was lower in NMO than in MS, indicating more severe axonal injury.[30–32] Additionally, retinal vascular changes, including attenuation of the peripapillary vascular tree and focal arteriolar narrowing, seem commoner in NMO.[33]

## Genetics

NMO is a sporadic disorder and familial cases are rare. The gene for AQP4 is located on 18q11.2-q12.1. Matiello *et al*. recently reported on 100 sporadic and 10 familial NMO cases (106 NMO-IgG seropositive); they detected mutations at Arg19 in 1.8% of NMO patients but not in control subjects.[34] These missense mutations occur within the 22 residues of genomic DNA that is unique to the AQP4 M1 isoform. Though it is the M23 isoform that is the target for the antibody, such mutations may confer M23-like properties on the mutant product.

## Pathogenesis of NMO

Up to 80% of patients with typical NMO have an antibody in their serum that seems to be highly specific for NMO. This antibody was originally described using immunofluorescence techniques at the Mayo Clinic and was named NMO IgG.[1,2] European and Asian groups have subsequently confirmed these findings and have developed other methods of detection.[15] The term ‘anti-aquaporin antibody’ (highlighting the antigenic target) is widely used outside of the Americas. Surprisingly, only one of the eight patients with NMO from a South Indian cohort with demyelinating disease was positive for NMO IgG.[35]

AQP4 antibody is produced by B cells in the peripheral circulation and access its antigenic target, the water channel AQP4, on astrocyte membranes. Regions with high expression of AQP4, such as the foot processes of astrocytes abutting the capillary walls in the blood–brain barrier, the optic nerve head, the spinal cord, and those regions where no blood–brain barrier exists (e.g., circumventricular organs), seem more susceptible to damage. After crossing the blood–brain barrier, binding occurs only to macromolecular aggregates of AQP4 (orthogonal array particles; OAP). Only the M23 isoform of AQP4 (and not M1) form such OAPS.[36] AQP4 and the sodium-dependent excitatory amino acid transporter-2 (EAAT2) coexist as a complex on the plasma membrane. EAAT2 is crucial to the reuptake of glutamate.[37] The binding of AQP4-Ab to AQP4 on astrocytes leads to the AQP4 being internalized by the cell [into early endosome antigen-1 (EEA1) containing early endosomal vesicles, with probable subsequent degradation[38–40]] along with EAAT2, resulting in impaired glutamate uptake and leading to excessive glutamate outside the cell. This, in turn, leads to injury to neurones and oligodendrocytes in the vicinity.[37] In addition, the water transport across astrocyte membranes is impaired due to functional impairment of AQP4. Granulocytes attracted by complement, null killer cells, and antibody-dependent cellular cytotoxicity, all contribute to further tissue injury.[40] It is plausible that upregulation of EAAT2 or prevention of OAP formation could limit injury in NMO.[37,41]

Tissue injury in NMO IgG–negative patients may be due to an as yet unidentified antibody[42] or may be mediated by mechanisms other than autoantibodies, e.g., cell-mediated cytotoxicity.[43]

## Clinical Utility of AQP4-Ab Testing

Discovery of a novel antibody always creates a stir in the scientific immunity. This is followed by the stages of scepticism, acceptance (when the test is externally validated), and increasing use (and misuse) until, finally, it is assigned its rightful place in clinical practice. NMO IgG/anti-AQP4 antibody seems to be in the throes of such changes. While it is not yet available in certain parts of the world, its use has been incorporated into a standard battery of tests for demyelinating diseases elsewhere. Though, ideally, any case of optic neuritis or transverse myelitis or an atypical brain MRI of potentially demyelinating diseases could be tested, the high costs and the poor availability of such testing mandates a pragmatic approach. Its utility in the *diagnosis* of clinically typical NMO is rather moot. Irrespective of the presence or absence of the antibody, management remains the same. However, it has an important role in cases where the diagnosis is uncertain (e.g., when MRI reveals a longish – but less than three segment long – demyelinating lesion, a first episode of severe optic neuritis or a longitudinally extensive myelitis). The identification of the AQP4-Ab in these clinical contexts would confirm the diagnosis of NMO spectrum disorder, and allow discussion with the patient regarding the possibility of future relapses and prophylactic immunotherapy. It is my practice to do antibody testing in such patients.

For whom should AQP4-Ab testing not be done? There seems to be no rationale in testing patients who have another obvious cause for the symptoms or have clinically definite NMO. A very short segment of myelitis, particularly if only the periphery of the cord is involved; an isolated episode of optic neuritis, which has shown good improvement, and those patients with the typical clinical and neurological findings of MS need not be tested.[44]

There are suggestions that the antibody levels follow clinical relapses. In a recent article AQP4–Ab levels and CD19 cells usually rose before a relapse and fell with treatment.[45] However, relapses did not always occur with high levels of antibody, indicating that the antibody alone is insufficient to induce relapses. All immunosuppressant medications, including steroids, seem to lower AQP4-Ab levels, while β-interferons do not. If confirmed, these observations can guide therapy, e.g., to preemptively treat potential relapses.

Three main laboratory techniques are utilized in identifying the antibodies[46] [Table 1]. The reported sensitivity and specificity of all three are broadly similar.[46]

**Table 1:** Sensitivity and specificity of anti-AQP4-Ab detection methods

Method	Sensitivity	Specificity
Indirect immunofluorescence (NMO-IgG)	86%	91%
Cell-based assays (Anti-AQP4-Ab)	91%	100%
Immunoprecipitation assays (anti-AQP4-Ab)	83%	100%

## Aquaporino ‘pathies’ in Neurological Disease

NMO IgG/anti-AQP4-Ab is the first and only antibody identified in inflammatory demyelinating disorders of the CNS with a specific target antigen. Since AQP4 is a water channel involved in the CNS transport of water, it may have a role in many disorders.

The posterior reversible encephalopathy syndrome (PRES) is said to be associated with NMO spectrum disorders.[47] The etiology of PRES is enigmatic, and a potential role for the AQP4 water channel in its pathogenesis is plausible. A recent study on a cohort of 70 consecutive NMO patients from the Mayo Clinic found five patients with PRES.[47] All were NMO antibody positive. Both PRES and NMO are rare disorders and the co-occurrence of the two in 7% of patients indicates that this co-occurrence may not be coincidental. Patients with NMO-PRES presented with chorea, altered sensorium, nystagmus, lethargy, aphasia or agitation, and cortical blindness. All recovered completely. A similar report in an Israeli cohort (where two out of five patients recovered) supports this report.[48] Alterations in transmembrane water flux due to AQP4 dysfunction may predispose to PRES. However, the exact mechanisms or the link to NMO is still unclear. In fact, 14 patients with PRES (without NMO) tested negative for the antibody.[47] Even if NMO IgG is relevant to the generation of PRES, it will account for only a minority of cases. The mechanisms of cellular edema following acquired brain injuries (for example head injury) and central pontine myelinolysis may involve AQP4.

One study found that nine of 12 NMO patients and seven of eight LETM patients had severe salivary gland inflammation (a hall mark of Sjögren syndrome).[49] AQP4 is expressed in low levels in salivary glands whereas AQP5, which shares 50% protein sequence with AQP4, is expressed at high levels and plays a major role in salivary gland secretion. It is plausible that patients with NMO may have a subset of autoreactive immune cells that recognize homologous portions of AQP4 and AQP5 and cause inflammation in both the CNS and the salivary glands.

Though NMO IgG is highly specific for NMO, it can also occur as a paraneoplastic phenomenon.[50] Of the 180,000 sera samples tested for paraneoplastic antibodies at the Mayo Clinic, 0.02% (*n* = 33) had NMO IgG and of these 93% (*n* = 26) had NMO or NMO spectrum disorders; 27% (*n* = 7) of these had cancer. Cancer preceded NMO in five patients and followed NMO in two (at 5 and 3 months after onset). The cancers detected affected breast, lung, and thyroid; one person had a lymphoma and another had a monoclonal gammopathy. Two patients who had NMO IgG, without signs of NMO/NMO spectrum disorder, had cancer.

## Treatment

In view of the antibody-mediated mechanisms underlying NMO it seems logical to treat the disorder with immunosuppressant medications. However, little high-quality evidence exists in support of the role of the various immunosuppressant therapies that are utilized.[3] The rarity of the disease, the severity of the relapses, and the early onset of morbidity and mortality make controlled trials difficult and placebo-controlled trials unethical.

Treatment of NMO involves acute treatment of relapses, prevention of relapses, symptom management, and rehabilitation. Management of relapses is with early institution of steroid treatment, typically 1 g of intravenous (IV) methylprednisolone for 5 days followed by oral prednisolone, starting with 1 mg per kg body weight and tapered over a 6–12 month period. Relapses that do not respond to IV steroids could benefit from plasma exchanges, typically seven exchanges over a 2-week period.[51–53] Remarkable recoveries have been noted to occur following plasma exchange.[54]

A steroid-sparing immunosuppressant agent is typically introduced soon after relapse, usually in hospital or during the first few weeks. Azathioprine is widely used, its popularity based only on a short case series,[55] convention, convenience, cost, and familiarity amongst neurologists. It is reasonably effective in most patients. Low-dose steroids,[56] methotrexate, cyclophosphamide, mitoxantrone and cyclosporin are other cheaper options and are supported by case reports.[3] Many patients relapse when attempts are made to withdraw steroids completely and it is reasonable to maintain such patients on a combination of azathioprine (or an equal) with the lowest possible dose of steroids (typically about 10–20 mg of steroid given on alternate days).

Rituximab, an anti-CD20 monoclonal antibody, has gained popularity following two case series that showed benefit in aggressive and otherwise therapy-resistant cases.[57,58] A retrospective multicenter case series of 25 NMO patients (including 2 children) treated with rituximab were followed up for a median of 19 months.[58] The median annualized post-treatment relapse rate was lower than the pretreatment rate [0 (range: 0–3.2) *vs* 1.7 (range: 0.5–5) relapses; *P*<0.001)]. Disability improved or stabilized in 20 of 25 patients (80%; *P* = 0.02). Two patients died during the follow-up period: one due to a brainstem relapse and the other owing to suspected septicemia. Infections were reported in 20% of patients.

Similar benefits were seen with mycophenolate in a 24-patient (seven of whom were treatment naïve) retrospective series.[59] At a median dose of 2000 mg/day and a median follow-up of 28 months (range 18–89) 19 (79%) patients remained on treatment. The median annualized post-treatment relapse rate was lower than the pretreatment rate [0.09 (range: 0–1.5) *vs* 1.3 (0.23–11.8); *P*<0.001]. Disability improved or stabilized in 22 out of 24 (91%) patients. One patient died during the follow-up period. Six (25%) patients noted side effects during treatment with mycophenolate.

It is likely that most immunosuppressants have a beneficial role in NMO. It is therefore important that cheaper conventional alternatives (like methotrexate, azathioprine, mitoxantrone, cyclosporine, and cyclophosphamide) are utilized when rituximab or mycophenolate are unavailable or when the high costs of newer therapies are prohibitive.

The treatment of NMO spectrum disorders (assuming that they usually follow the clinical course of NMO) should logically be similar to that of NMO. The probability of permanent disability with each relapse persuades many clinicians to offer, and patients to opt for, immunotherapy.

There are many unanswered questions in NMO. Animal models have not yet successfully reproduced illness that is pathologically similar to human disease. Though AQP4 is expressed in kidneys, lungs, inner ear, muscle, and intestines and these organs are more accessible to the antibody, why do they remain unaffected in NMO? Why does the deletion of AQP4 gene in mouse models not cause NMO-like phenotypes?[60] What causes the breach in the blood–brain barrier, allowing initial entry of the antibody into the CNS? What is the disease mechanism in the 20–30% of patients with typical NMO who have no identifiable antibody? And what about the as yet unsettled debates on the opticospinal Asian MS and NMO? Are they *really* the same?[61,62]

## Conclusions

Exciting developments have occurred in the last two years in NMO. Our understanding of the mechanisms of the disease has expanded rapidly, perhaps even more so than in MS. However, improvement in patient outcomes will still depend on early diagnosis, aggressive management of relapses, and early effective immunosuppression. It has to be emphasized that disability is acquired within the first few relapses itself. It is also important to remember that commonly available and cheap immunosuppressants could be as effective as newer, more expensive, agents.
